# Assessing the costs and efficiency of HIV testing and treatment services in rural Malawi: implications for future “test and start” strategies

**DOI:** 10.1186/s12913-020-05446-5

**Published:** 2020-08-12

**Authors:** Seema Vyas, John Songo, Lorna Guinness, Albert Dube, Steffen Geis, Thokozani Kalua, Jim Todd, Jenny Renju, Amelia Crampin, Alison Wringe

**Affiliations:** 1 Department of Population Health, London School of Hygiene and Tropical Medicine, Keppel Street, London, WC1E 7HT UK; 2 Malawi Epidemiology and Intervention Research Unit, Lilongwe, Malawi; 3 Independent researcher, Oxford, UK; 4 Institute of Medical Microbiology and Hygiene, Philipps University Margburg, Marburg, Germany; 5 Department of HIV and AIDS, Ministry of Health, Lilongwe, Malawi

**Keywords:** Malawi, HIV testing services, HIV care and treatment, Costs, Financing, efficiency

## Abstract

**Background:**

Reaching the 90–90-90 targets requires efficient resource use to deliver HIV testing and treatment services. We investigated the costs and efficiency of HIV services in relation to HIV testing yield in rural Karonga District, Malawi.

**Methods:**

Costs of HIV services were measured over 12 months to September 2017 in five health facilities, drawing on recognised health costing principles. Financial and economic costs were collected in Malawi Kwacha and United States Dollars (US$). Costs were calculated using a provider perspective to estimate average annual costs (2017 US$) per HIV testing episode, per HIV-positive case diagnosed, and per patient-year on antiretroviral therapy (ART), by facility. Costs were assessed in relation to scale of operation and facility-level annual HIV positivity rate. A one-way sensitivity analysis was undertaken to understand how staffing levels and the HIV positivity rate affected HIV testing costs.

**Results:**

HIV testing episodes per day and per full-time equivalent HIV health worker averaged 3.3 (range 2.0 to 5.7). The HIV positivity rate averaged 2.4% (range 1.9 to 3.7%). The average cost per testing episode was US$2.85 (range US$1.95 to US$8.55), and the average cost per HIV diagnosis was US$116.35 (range US$77.42 to US$234.11), with the highest costs found in facilities with the lowest daily number of tests and lowest HIV yield respectively. The mean facility-level cost per patient-year on ART was approximately US$100 (range US$90.67 to US$115.42). ART drugs were the largest cost component averaging 71% (range 55 to 76%). The cost per patient-year of viral load tests averaged US$4.50 (range US$0.52 to US$7.00) with cost variation reflecting differences in the tests to ART patient ratio across facilities.

**Conclusion:**

Greater efficiencies in HIV service delivery are possible in Karonga through increasing daily testing episodes among existing health workers or allocating health workers to tasks in addition to testing. Costs per diagnosis will increase as yields decline, and therefore, encouraging targeted testing strategies that increase yield will be more efficient. Given the contribution of drug costs to per patient-year treatment costs, it is critical to preserve the life-span of first-line ART regimens, underlining the need for continuing adherence support and regular viral load monitoring.

## Background

Renewed global efforts are being made to bring the HIV epidemic under control, notably through the release of the UNAIDS 90–90-90 targets in 2014 [1], and the subsequent World Health Organization (WHO) recommendations for universal “Test and Start” for HIV in 2015 [2]. As a result, the number of people on antiretroviral therapy (ART) in eastern and southern Africa reached 11.7 million in 2016, representing 60% of all people living with HIV (PLHIV) in the region [3].

At the individual level, the benefits of timely initiation of ART among those who test HIV positive include improved life expectancy and lower risks of opportunistic infections [4]. At the population level, the benefits of achieving the 90–90-90 targets would include reduced HIV transmission, including of drug-resistant HIV strains, in turn reducing the social and economic burden of HIV [5, 6]. Despite the urgency of achieving these health and development advantages, global financing for HIV has levelled since the global economic crisis of 2008–09 [7]. Innovations in HIV financing and increased domestic funds are being sought both nationally and internationally [8–10]. It has also been argued that more efficient use of existing resources, through regular resource allocation assessments, would make an essential contribution to addressing the funding gap [3, 11].

To understand the affordability of HIV services requires an assessment of its costs which is critical to estimating budgetary requirements. At the same time, these data contribute to the understanding of how to improve planning and allocate existing resources to maximise value for money or efficiency. While large scale, multi-site cost data are generally required to capture the variability in costs and efficiency [12], information on HIV service costs in sub-Saharan Africa are limited [13]. Moreover, few analyses exist of how costs might change as the epidemic and corresponding care and treatment strategies evolve.

In Malawi, the HIV epidemic remains generalised with an estimated adult HIV prevalence of 10.6% by 2016 [14]. Seventy-three percent of PLHIV in Malawi self-report knowing their HIV status (67% among men and 77% among women), 89% of whom are on ART of which 91% are virally suppressed [14]. These achievements in testing and treatment coverage reflect progress in the national scale-up of HIV testing services and HIV care and treatment, a process which accelerated in 2011 with the introduction of Option B+, lifelong HIV treatment for all pregnant women living with HIV, and subsequent adoption of Treat-all policies in 2016 [15].

While these achievements are impressive, new strategies, including better targeting of HIV testing and care and treatment, will be needed to achieve the 90–90-90 targets in Malawi. However, further expansion of targeting HIV services is dependent on resource availability. Assessing the resource requirements and therefore, affordability, requires an understanding of the costs of service delivery, data which can also help to ensure better and more efficient use of existing resources and to improve planning and budgeting. To date, cost studies in Malawi have exclusively examined either the costs of HIV testing or the costs of ART, or have estimated costs at a time when previous HIV policy guidelines were implemented (Table s1) [16–18]. As such, research is needed that provides facility-based costs of HIV testing and care and treatment services in the context of universal test and treat, particularly in rural areas which typify the context of most HIV treatment programmes. In this context, the aim of this study was to assess how facility characteristics and HIV testing positivity rate impacts on costs and efficiency of HIV services in a rural district in northern Malawi.

## Methods

### Facility-based HIV services in Malawi

In 2016, the Ministry of Health (MoH) in Malawi released updated guidelines on HIV testing and on the clinical management of HIV in adults and children in efforts to move closer to its goal of achieving universal access to ART [19, 20]. The MoH, supported by implementing partners, works with its regional and district health offices to provide HIV prevention and treatment services in accordance with these guidelines (Table s2). These services are supported by a number of national and district-level activities including surveillance and auditing visits, quality assurance of services, and monitoring and evaluation (M&E) activities which involves the routine collection, recording, analysis, and reporting of Health Management Information Systems (HMIS) data. The HMIS is a national database of records on key health indicators, including on HIV, for each facility.

### Study setting

Malawi is divided into three regions (Central; Northern and Southern Regions) which are further sub-divided into a total of 28 districts. Karonga District, Northern Region, was selected for this study because it hosts a health and demographic surveillance site (HDSS) in the southern part of the District. The HDSS continuously monitors demographic events among a population of approximately 40,000 residents through annual surveillance rounds [21]. Since 2013, bi-annual health facility surveys have been conducted in all facilities (*n* = 5) providing HIV services to the HDSS population in order to assess HIV service delivery [22], although the HDSS does not offer any service support to these facilities. The third round additionally included a nested costing study. In 2015–16, HIV prevalence among adults (ages 15–49 years) was estimated at 8.7% (in males) and 10.5% (in females) in Karonga District [23].

### Facility characteristics

All five HDSS health facilities were included in this study. Three facilities are classified as clinics (one small and two large) and two facilities are classified as a hospital (one small and one medium size) (Table 1). Two clinics (Clinics A and C) have a delivery ward. Three facilities are government-run (public) and two facilities are faith-based.

**Table 1:** Facility characteristics [source facility survey; HMIS]

Facility	Size/type^a^	Authority	Number outpatient attendance^b^	Location
Clinic A	Small clinic	Faith-based	3437	Remote rural
Clinic B	Large clinic	Public	25,451	Rural
Clinic C	Large clinic	Public	17,778	Remote rural
Hospital D	Small hospital	Faith-based	5464	Rural
Hospital E	Medium hospital	Public	36,535	Rural

^a^Facility type classified by facility in-charge. Size classified by authors and is based on number of outpatient visits i.e. small clinic & small hospital < 10,000 outpatient visits per annum

^b^Annual outpatient attendance from October 2016 to September 2017

### Cost study

The methodology for this cost study drew on costing guidelines for HIV prevention strategies and on the principles of the “Reference case for global health costing, 2017”, a guide developed to assist researchers in the process of generating transparent and comparable cost estimates [24, 25].

This study was undertaken from the health providers’ perspective (governments and donors), and estimates the cost of core facility-based HIV testing and treatment services. Costs include those associated with activities which occur within each facility, and those that are managed at the district and the national level and which are essential to the delivery of the service (Table s2).

HIV testing services, defined as facility-based testing using rapid HIV tests, includes the provision of both pre- and post-test counselling, first test and confirmatory testing, and training of health care workers on HIV testing. HIV treatment services include the provision of antiretroviral medicines according to the 2016 national guidelines, Cotrimoxazole preventive therapy, viral load laboratory tests, patient monitoring, and the training of health workers on care and treatment. There are five standard first-line ART regimens, of which two fixed-dose combinations (one adult and one paediatric) are used for ART initiation, and three standard second-line ART regimens (Table s3) [20]. Isoniazid preventive therapy is not included because Karonga District is not classified as a high-risk tuberculosis area [19]. Within the treatment protocol patients make regular visits to clinics to collect ART drugs and for other treatments, counselling and viral load testing [20].

### Data collection

A standardised Microsoft Excel-based instrument was used to record resource use and, where available, price information from each facility. Data were collected retrospectively for the 12 months to September 2017. Most data collection took place during October and November 2017. Resources were categorised into capital inputs (buildings/room space, equipment, and staff training) and recurrent inputs (personnel, HIV test kits, ART, Cotrimoxazole, viral load tests, other clinical and non-clinical supplies, overheads, and national/district supervision, auditing, and M&E visits).

*Service delivery data* were captured from two sources 1) HMIS; and 2) from facility records (registers, summary reports, and ART patient cards). Routine data on persons tested for HIV were extracted from facility registers. Data extracted from ART patient cards were ART regimen and number of tablets by month of visit. In facility E, patient utilisation data were obtained from monthly summaries compiled by clinical staff.

### Cost approach

Two types of costs were estimated: 1) financial costs, defined as the actual expenditure incurred on resource inputs (salaries, HIV test kits, ART, Cotrimoxazole and other HIV supplies), and 2) economic costs, defined as the value of all resource inputs, including donated or subsidised items, which were valued at their market price (rent, capital equipment and volunteer time). A micro-costing approach, a method which identifies and values each input consumed in delivering a service [25], was applied which used a combination of top-down and bottom-up approaches to obtain resource use and costs depending on the line item.

*Capital inputs:* Physical space and office equipment used to deliver HIV services was audited and measured during visits to each facility. Shared usage across testing and treatment services was identified from interviews with staff. The cost of space used was estimated from rental costs for equivalent nearby spaces. Prices for office equipment (e.g. furniture and medical equipment) were obtained from the Karonga District Health Office and from medical suppliers in the country. Capital equipment prices were annualised over 25 years. Training costs, provided to health workers in the previous 2 years, were estimated using information provided by the District Health Office, (staff per diems and travel expenses) and by national non-governmental organisations running HIV training programs (unit “per participant” training costs). Staff training costs were annualised over 2 years, in line with MoH guidelines that all registered/certified HIV service providers are to receive training at 2 year intervals [19, 20].

*Recurrent inputs:* Staff delivering HIV services were interviewed to illicit the amount of time that workers spent on different HIV services and salaries were apportioned accordingly. Salaries (wages and benefits) were collected from either the facilities (two faith-based facilities) or from the District Health Office. Per diems for lay counsellors were collected from the staff.

Prices of HIV test kits and other clinical and non-clinical supplies (including national HIV testing registers and HIV patient treatment cards) were obtained from medical suppliers based in Lilongwe. Drug costs (ART and Cotrimoxazole) were obtained from The Global Fund ART pricing list for 2017 [26]. Laboratory costs of viral load tests were obtained from organisations providing the service, and the cost of reagents etc. from medical supplies organisations. Overhead costs included the cost of utilities (electricity, water, buildings and equipment maintenance, telecoms, waste water, and administration in Clinic B and both hospitals, and electricity, water and telecoms in Clinics A and C). Overheads costs were apportioned to the HIV service based on the size of space used to deliver the service. Facility audits, supervision and M&E cost information (salaries, allowances, accommodation, transport expenses, and airtime) were provided by the MoH and by the Karonga District Health Office.

### Cost analysis

Facility survey data were analysed using STATA v15. Facility-level cost and outcome calculations were undertaken using Microsoft Excel 2007. Total annual costs and average (unit) costs were calculated for each facility and separately for HIV testing and for HIV care and treatment services. All costs were adjusted to 2017 United States dollars (US$) using an average exchange rate over the 12 month study timeframe (1 US$ = 724.35 Malawi Kwacha) [27]. Because all costs were collected in current prices, adjustment for inflation was not required.

The levels of technical efficiency (relationship between inputs to the service outputs) and economic efficiency (relationship between the monetary value of the inputs to the service outputs) of HIV testing and HIV care and treatment were estimated as:

1. *HIV testing services:* The cost per testing episode (full testing procedure for an individual and which may include confirmatory testing) and per HIV case detected were calculated by dividing the total facility costs of running testing services in 1 year by the number of testing episodes, and the number of new HIV-positive individuals identified over the same timeframe (annual HIV positivity rate).
2. *HIV care and treatment:* The annual cost per patient-year for ART care was calculated by dividing the total annual costs of providing care and treatment services by the number of HIV patients receiving care and treatment.

A one-way sensitivity analysis, varying one parameter at a time holding all others constant, was undertaken to understand how staffing levels and the annual HIV positivity rate affected the costs of HIV testing services. Cost variations were explored by applying a +/− 10% variation range to staffing levels and the annual positivity rate, and by applying annual HIV positivity rates observed in studies from other settings in Malawi.

## Results

### HIV testing service delivery and costs

The mean number of health workers who provided HIV testing services across the five facilities was 7.4 (range 3 to 13) yielding an average of 3.3 full time equivalent (FTE) per facility (total 16.3 FTE; range 1 to 4.6 FTE), of which approximately 3.0 (total 15.1; range 0.6 to 4.0 FTE) were counsellors (professional and lay) (Tables 2 & s4).

**Table 2:** Number of HIV tests conducted from October 2016 to September 2017

Facility	No. staff / trained	No. FTE staff	No. FTE counsellors^a^	Testing episodes *(1st test Determine)*	Confirmatory *(2nd test Unigold)*	New positive	Annual positivity rate
Clinic A	4 / 3	1.0	0.6	520	26	19	3.7%
Clinic B	9 / 9	4.0	4.0	3144	178	74	2.4%
Clinic C	3 / 0	2.5	2.5	1916	64	36	1.9%
Hospital D	13 / 0	4.2	4.0	3385	152	84	2.5%
Hospital E	8 / 0	4.6	4.0	9544	553	240	2.5%
***Total***	***37 / 12***	***16.3***	***15.1***	***18,509***	***973***	***453***	
***Average***	***7.4 / 2.4***	***3.3***	***3.0***	***3702***	***195***	***91***	***2.4%***

^a^Includes lay (volunteer) counsellors: 2 in Clinic C

A total of 12 health workers were trained in HIV testing in the past 2 years (nine from Clinic B, three from Clinic A). No health workers were trained in HIV testing during these 2 years at the other facilities. HIV testing surveillance or audits were conducted on a quarterly basis in all five facilities, and supervision visits were conducted every month.

Over the 12 months to September 2017, a total of 18,509 HIV testing episodes (1st tests, 2nd tests and confirmatory) were conducted. The mean number of testing episodes per facility was 3702 (range 520 to 9544). The average number of new HIV positive cases across all five facilities was 91 (total 453; range 19 to 240), yielding an average annual HIV positivity rate of 2.4% (range 1.9 to 3.7%).

**Fig. 1:**
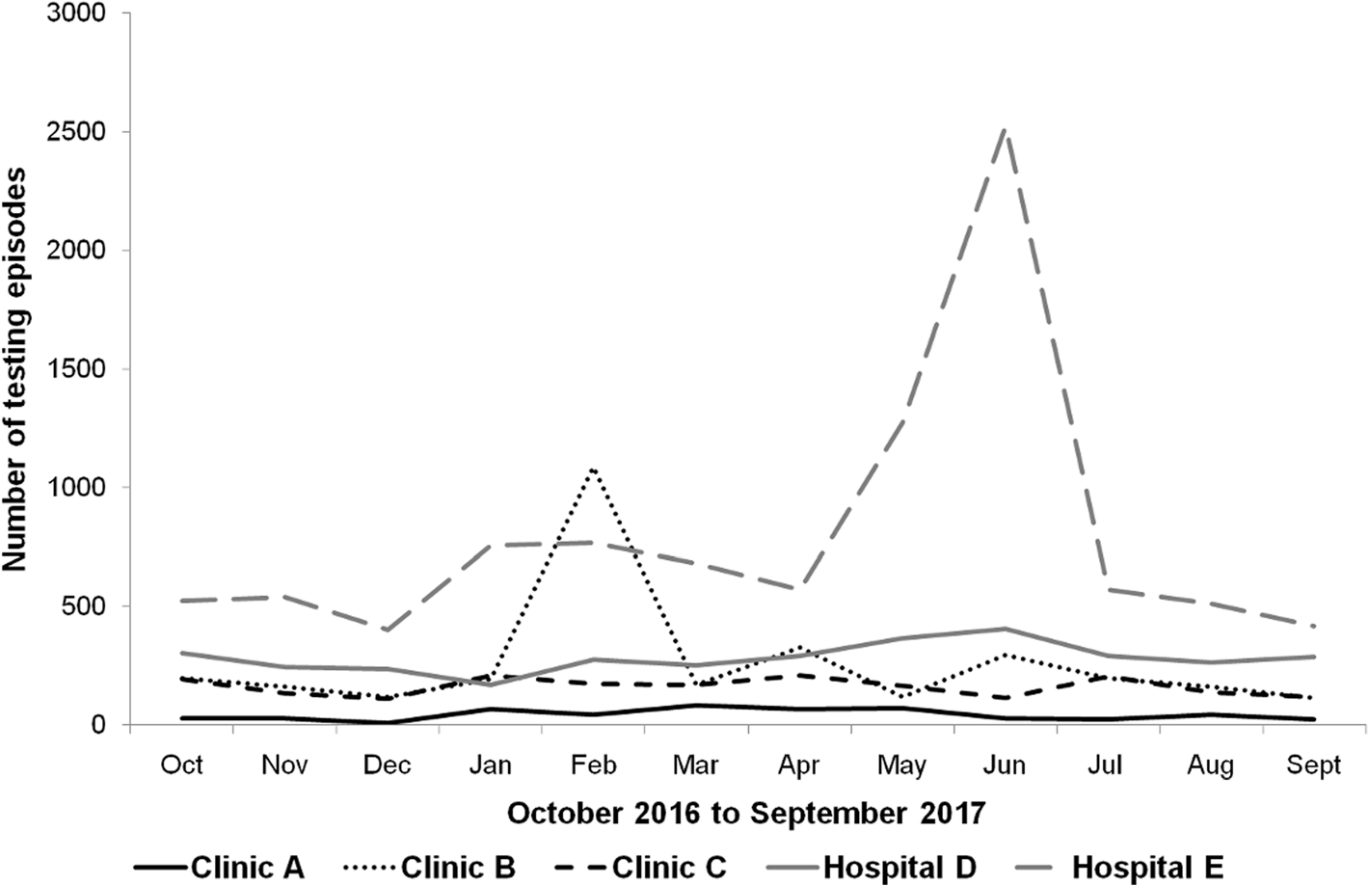
Number of HIV testing episodes by health facility and by month

Based on the number of days per week that facilities conducted HIV tests (7 days Hospital E, 6 days Hospital D, and 5 days in the three clinics), the number of HIV testing episodes conducted per day and per FTE HIV testing staff averaged 3.3 (range 2.0 Clinic A to 5.7 Hospital E). No clear trend was observed over the 12-month period in the number of HIV tests undertaken by month and per facility (Fig. 1). In facilities B and E, testing activity peaked in February and June respectively.

The total annual cost of HIV testing in the facilities ranged from $4448 (Clinic A) to $18,582 (Hospital E) (Table 3). The cost per testing episode averaged $2.85 (range $1.95 Hospital E to $8.55 Clinic A). There was also variation in the cost per new HIV positive case detected across the facilities which averaged $116.35 (range $77.42 Hospital E to $234.11 Clinic A). There was an inverse relationship between the number of testing episodes and the cost per testing episode, however, no clear trend was observed between the cost per person diagnosed and facility annual HIV-positive yield.

**Table 3:** Annual HIV testing services costs; cost per testing episode (cost per test); and cost per HIV case diagnosed (cost per HIV) in 2017 US$ (October 2016 to September 2017)

	Clinic A			Clinic B			Clinic C			Hospital D			Hospital E		
	Total cost	Cost per test	Cost per HIV	Total cost	Cost per test	Cost per HIV	Total cost	Cost per test	Cost per HIV	Total cost	Cost per test	Cost per HIV	Total cost	Cost per test	Cost per HIV
**CAPITAL COSTS**															
Building	115	0.22	6.04	14	0.00	0.19	22	0.01	0.60	26	0.01	0.30	215	0.02	0.90
Equipment	15	0.03	0.80	78	0.02	1.05	13	0.01	0.36	65	0.02	0.77	33	0.00	0.14
Training	393	0.76	20.69	1179	0.38	15.94	0	0.00	0.00	0	0.00	0.00	0	0.00	0.00
**Total capital**	**523**	**1.01**	**27.53**	**1272**	**0.40**	**17.19**	**35**	**0.02**	**0.96**	**90**	**0.03**	**1.08**	**249**	**0.03**	**1.04**
**RECURRENT COSTS**															
Personnel	2109	4.06	111.02	5116	1.63	69.13	4615	2.41	128.21	7154	2.11	85.17	7537	0.79	31.40
HIV test kits	458	0.88	24.08	2800	0.89	37.84	1821	0.95	50.59	2954	0.87	35.17	8531	0.89	35.55
Other supplies	179	0.34	9.43	525	0.17	7.09	152	0.08	4.22	635	0.19	7.56	842	0.09	3.51
Utilities & other	433	0.83	22.82	81	0.03	1.09	35	0.02	0.98	155	0.05	1.84	678	0.07	2.82
**Total recurrent**	**3180**	**6.11**	**167.35**	**8521**	**2.71**	**115.15**	**6624**	**3.46**	**183.99**	**10,898**	**3.22**	**129.74**	**17,587**	**1.84**	**73.28**
**DISTRICT/NATIONALCOSTS**															
Supervision	410	0.79	21.55	410	0.13	5.53	410	0.21	11.38	410	0.12	4.88	410	0.04	1.71
M&E	336	0.65	17.69	336	0.11	4.54	336	0.18	9.33	336	0.10	4.00	336	0.04	1.40
**Total District/National**	**746**	**1.43**	**39.24**	**746**	**0.24**	**10.07**	**746**	**0.39**	**20.71**	**746**	**0.22**	**8.88**	**746**	**0.08**	**3.11**
**TOTAL/ AVERAGE**	**4448**	**8.55**	**234.11**	**10,538**	**3.35**	**142.41**	**7404**	**3.86**	**205.66**	**11,734**	**3.47**	**139.69**	**18,582**	**1.95**	**77.42**

**Fig. 2:**
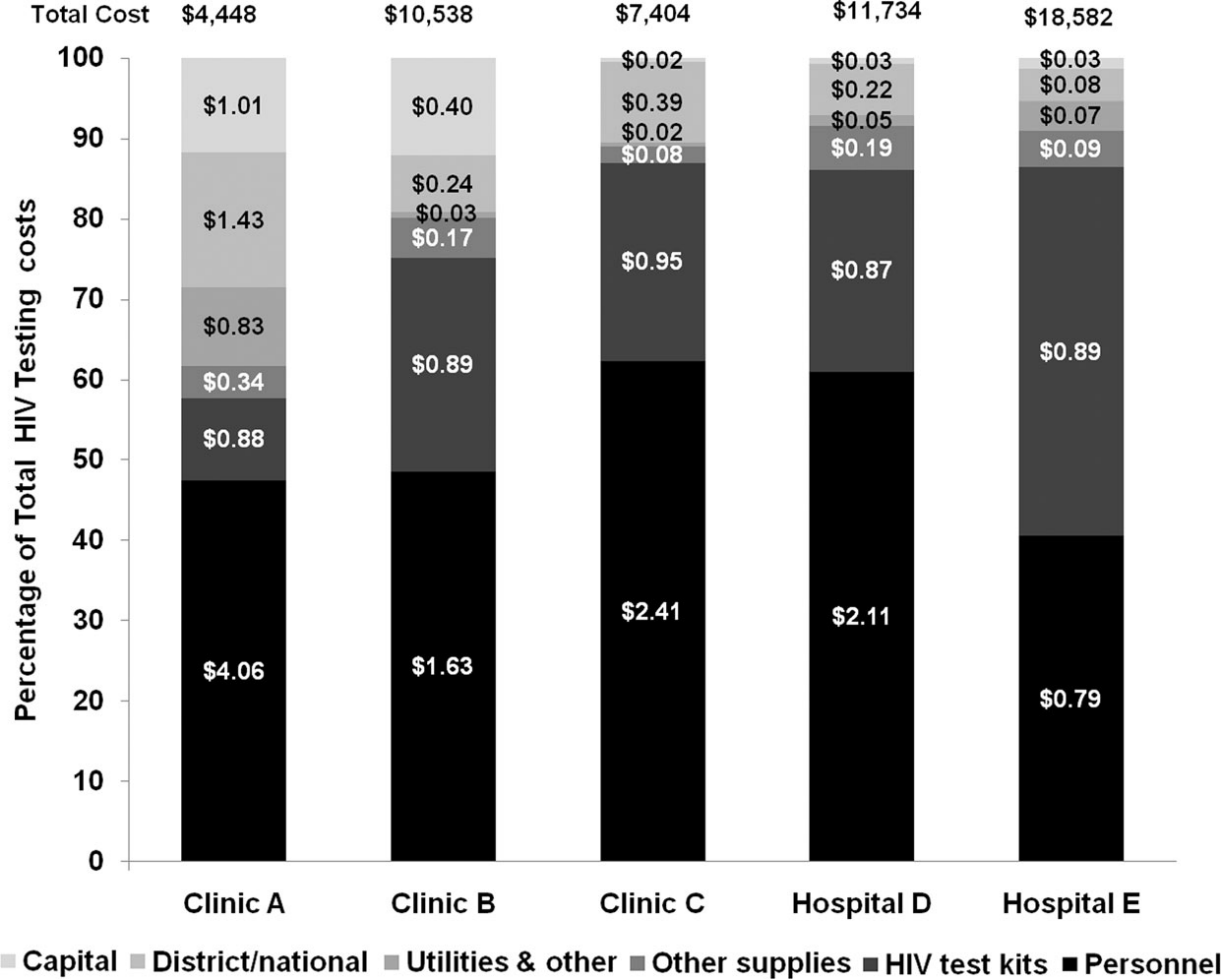
HIV testing resource input share of total HIV testing costs, and average cost per testing episode in US$. ($ figure overlaid in shaded bars is average cost per testing episode for resource input category)

Recurrent costs, in particular personnel followed by the cost of test kits, comprised the majority of total HIV testing costs (Fig. 2). Health worker time represented 50.0% of all HIV testing costs (range 40.6% Hospital E to 62.3% Clinic C). HIV test kits accounted for 31.4% of all testing costs (range 10.3% Clinic A to 45.9% Hospital E). National and district-level service delivery costs (supervision, audits and M&E), accounted for 7.1% of HIV testing costs (range 4.0% Hospital E to 16.8% Clinic A).

### Sensitivity analysis

When staff inputs were varied by +/− 10%, the mean cost per person diagnosed with HIV changed by +/− 5% and ranged from $110.49 to $122.20 (Table 4). Varying the annual positivity rate by +/− 10% resulted in a +/− 11% change in the mean cost per person diagnosed; range $105.77 to $129.28. Applying an annual positivity rate of 9%, as observed elsewhere in Malawi, reduced the mean cost per person diagnosed by 73% to $31.64.

**Table 4:** Sensitivity analysis results and percent change in cost in HIV positive person diagnosed

Parameter	Range from base estimate	2017 US$		% Change in cost
Staff level	(−10% to + 10%)	110.49	122.20	+/− 5%
Annual positivity rate	(−10% to + 10%)	129.28	105.77	+/− 11%
Annual positivity rate^a^	9%	31.64		− 73%

^a^As observed elsewhere in Malawi by Mwenge et al. [16]

**Fig. 3:**
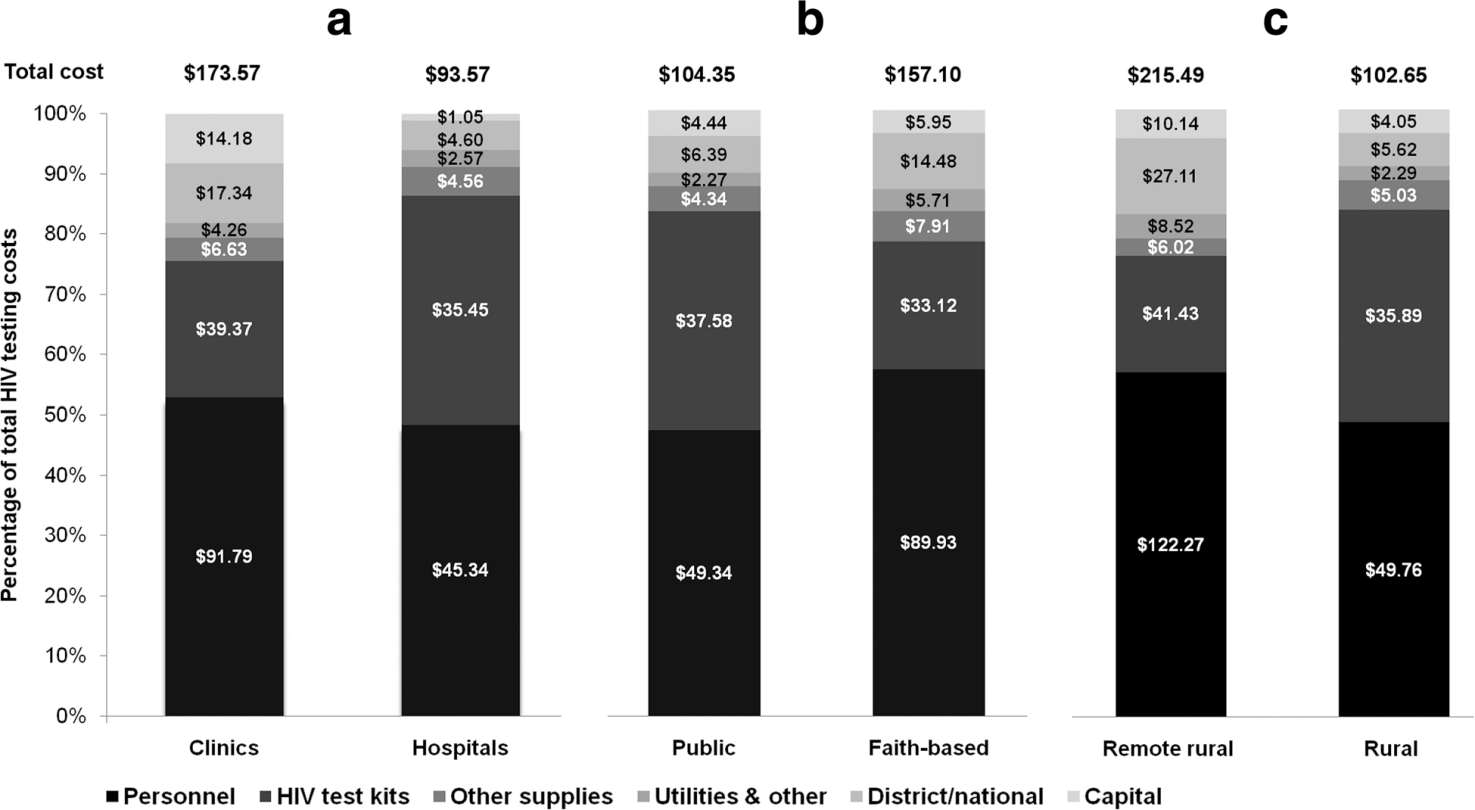
HIV testing resource input share of total HIV testing costs, and average cost per HIV case detected in $US in **a** Clinics versus hospital. **b** Public versus faith-based facilities. **c** Remote rural versus rural. ($ figure overlaid in shaded bars is average cost per HIV case diagnosed for resource input category)

When stratified by facility type, authority and location, higher mean costs per HIV case identified were observed in clinics versus hospitals (Fig. 3a), faith-based facilities versus public facilities (Fig. 3b), and remote rural versus rural facilities (Fig. 3c). The cost per person diagnosed was highest in remote rural facilities (US$215.49), and lowest in hospitals (US$93.57). The annual HIV positivity rate, however, varied very little across the strata (Table s5)

### HIV treatment service delivery and costs

The mean number of health workers who provided ART services was 8.4 per facility (total 42; range 2 to 17), and the mean number of FTE per facility was 2.5 (total 12.7 FTE; range 0.4 to 4.5 FTE) (Tables 5 & s4). Sixty-nine percent (29 of 42 of health workers who provided care and treatment had received ART training in the previous 2 years. All health workers providing care and treatment in each of the three clinics and in Hospital D had been trained in ART, compared with 23.5% (4 of 17) health workers who were trained in Hospital E. In all five facilities, national-level supervision visits for care and treatment services were reportedly conducted every quarter and district-level auditing was undertaken every month.

**Table 5:** HIV care and treatment service delivery (October 2016 to September 2017)

Facility	No. ART staff / trained	No. FTE ART staff	No. ART patients	No. ART adult/paediatric	No. viral load tests	No. initiated in past 12 months
Clinic A	3 / 3	0.4	187	178/9	7	13
Clinic B	10 / 10	4.5	477	459/18	133	83
Clinic C	2 / 2	1.0	233	218/15	80	40
Hospital D	10 / 10	2.4	192	185/7	96	53
Hospital E	17 / 4	4.4	1021	952/69	362	223
***Total***	***42 / 29***	***12.7***	***2110***	*1992 / 118*	***678***	***412***
***Average***	***8.4 / 5.8***	***2.5***	***422***	***398 / 24***	***136***	***82***

The number of ART current patients totalled 2110 across the five facilities (range 187 to 1021). The total number of newly initiated ART patients over the 12 months was 412 (range 13 to 223), which equates to 91% of the new HIV-positive cases (*n* = 453) identified over the timeframe. The total number of viral load tests was 678 (range 7 to 362).

The proportion of patients on paediatric ART averaged 5.6% (range 3.6 to 6.8%), and all were on the first-line 3TC/AZT/NVP regimen. Among the 1992 adult ART patients, 92.4% were on the first-line regimen TDF/3TC/EFV 300/300/600, with a further 5.6% on a different first-line regimen. Only 2% of adult ART patients were on second-line regimens. On average, ART was dispensed to patients every 2.9 months or 4.1 times per patient-year (range every 2.6 to 3.2 months, or 3.8 to 4.7 times per patient-year).

The total annual HIV care and treatment costs ranged from $19,003 (Clinic A) to slightly over $100,000 (Hospital E) (Table 6). The mean facility-level care and treatment cost per patient-year was almost $100 ($99.35); range $90.67 (Clinic C) to $115.42 (Hospital D).

**Table 6:** Annual HIV care and treatment costs; and cost per patient-year on ART in 2017 US$ (October 2016 to September 2017)

	Clinic A		Clinic B		Clinic C		Hospital D		Hospital E	
	Total cost	Cost per ART patient	Total cost	Cost per ART patient	Total cost	Cost per ART patient	Total cost	Cost per ART patient	Total cost	Cost ART per patient
**CAPITAL COSTS**										
Building	555	2.97	16	0.03	23	0.10	5	0.03	239	0.23
Equipment	17	0.09	129	0.27	52	0.22	13	0.07	53	0.05
Training	786	4.20	2621	5.49	524	2.25	2621	13.65	1048	1.03
**Total Capital**	**1358**	**7.26**	**2621**	**5.49**	**599**	**2.57**	**2639**	**13.74**	**1341**	**1.31**
**RECURRENT COSTS**										
Personnel	850	4.55	6668	13.98	1785	7.66	4046	21.07	9411	9.22
ART	12,610	67.43	32,270	67.65	15,482	66.45	12,266	63.89	76,619	75.04
Cotrimoxazole	1098	5.87	2710	5.68	1324	5.68	1061	5.53	6241	6.11
Viral load	98	0.52	1862	3.90	1120	4.81	1344	7.00	5068	4.96
Other supplies	24	0.13	64	0.13	31.34	0.13	27	0.14	142	0.14
Utilities & other	2220	11.87	86	0.18	38.58	0.17	32	0.17	752	0.74
**Total Recurrent**	**16,899**	**90.37**	**43,661**	**91.53**	**19,781**	**84.90**	**18,776**	**97.79**	**98,233**	**96.21**
**DISTRICT/NATIONAL COSTS**										
Supervision	410	2.19	410	0.86	410	1.76	410	2.13	410	0.40
M&E	336	1.80	336	0.70	336	1.44	336	1.75	336	0.33
**Total District/National**	746	3.99	746	1.56	746	3.20	746	3.88	746	0.73
**TOTAL / AVERAGE**	**19,003**	**101.62**	**47,027**	**98.59**	**21,126**	**90.67**	**22,160**	**115.42**	**100,319**	**98.26**

Recurrent costs contributed the most to total costs, of which ART drugs were the largest cost component averaging 71% across the five facilities (range 55% Hospital D to 76% Hospital E). This translated to an average ART drug cost of $70.73 US per patient-year across the five facilities (range $63.89 to $75.04). Cotrimoxazole drug costs contributed, on average, a further 6% to total costs, or $5.89 per patient-year. Personnel comprised the second largest cost component (after drugs) averaging 11% (range 4 to 18%) or $10.79 (range $4.55 to $21.07).

The variability in the average annual per patient cost of viral load laboratory tests, which ranged from $0.52 (Clinic A) to $7.00 (Hospital D), reflected the differences across the facilities in the ratio of viral load test per ART patient over the 12 months which ranged from a low of 4% (Clinic A) to 50% (Hospital D).

Per patient-year costs of capital inputs averaged $4.06 (4% of total costs) across the facilities; range $1.31 (Hospital E) to $13.74 (Hospital D) driven by the cost of ART training. National and district supervision costs averaged $1.77 per patient-year or 2% of total HIV care and treatment costs.

## Discussion

This district-level case study, to cost the delivery of facility-based HIV services in the context of Treat-All, highlights potential areas for cost-saving and has implications for future resource allocation in terms of how these services are delivered in this setting. These findings are particularly timely, given the current flat-lining of external funding and the imminent approach of the deadline for the 90–90-90 targets, and indicate key areas for strategy reform in HIV testing.

HMIS data reveal that the average annual HIV positivity rate has been declining year on year from 4.4% (2014) to 2.0% (2018) in these five facilities, suggesting that identifying new HIV cases through existing facility-based approaches may be close to reaching saturation. Our estimate of the mean cost per HIV case diagnosed (which ranged from US$77.42 to US$234.11) is higher than documented elsewhere in Malawi, and is likely driven by the comparatively lower HIV prevalence found in the area [15, 16]. Our estimate of the mean cost per testing episode (which ranged from US$1.95 to US$8.55) is comparable to cost estimates from southern and central Malawi and from Zambia, implying that the cost per HIV case diagnosed is driven by the HIV positivity rate [16, 17].

While facility-based testing has been a mainstay of Malawi’s achievement in diagnosing 73% of PLHIV by 2016, our findings suggest refocusing resources to differentiated HIV testing services to identify PLHIV who do not know their status. This would increase the diagnosis rate and reduce the cost per diagnosis. Alternative testing strategies such as home- and community-based testing, HIV self-testing, and index tracing are likely to become more necessary for diagnosing first-time testers and the hardest-to-reach persons including men and key populations [28, 29].

Our findings also confirm that as the facility-based HIV positivity rate declines and the cost per HIV diagnosis rises, staff time becomes an increasingly important contributor to costs. With the number of HIV testing episodes per FTE health worker per day well below the national guideline of a maximum of 15 per day, our results support policies to phase out the use of dedicated HIV counsellors in facilities, and to move towards greater role diversity among existing health workers providing other HIV or general health services. This recommendation, however, should take into account the numbers and levels of different health workers within a facility.

We estimated the average per patient-year cost of core HIV treatment services to be within a relatively narrow range across the facilities (US$90.67 to US$115.42), supporting the existing centralisation policies of ART delivery in small rural clinics. Seventy-one percent of annual treatment costs was ART drugs, which is similar to findings elsewhere in Malawi and in other sub-Saharan African countries [18]. Preserving the life-span of cheaper first-line ART drugs becomes increasingly important as moving to more expensive second-line drugs will cause the overall annual care and treatment cost to rise. This underlines the need for better coverage with viral load monitoring to identify treatment failure, adherence challenges, and to have a sufficient mechanism in place for prescribing second line treatment. However, our study findings indicate that current viral load testing coverage in these facilities does not meet national guidelines, which states that viral load testing should be done at 6 months and 2 years after initiation and then every 2 years thereafter [20]. Although increasing the number of viral load tests, in line with guidelines, will increase the cost of care, this gap between guidelines and the actual implementation of testing needs attention.

Limitations to this study include not costing some supporting HIV service components, including community-based testing, early infant diagnosis, or facility-based index testing. However, use of these testing mechanisms was very low during the study period. With respect to HIV treatment, we did not include the management and treatment of HIV-related illnesses, prevention services (such as the provision of insecticide-treated nets; family planning; or post-exposure prophylaxis) or home visits for patients who miss appointments. Further, we did not include patient costs which may act as a barrier to care. These components should be considered in future cost studies to ensure more holistic estimates of HIV service delivery costs. A second limitation, as found elsewhere [30], was that record-keeping across the facilities varied, which meant that some input prices, typically overheads, had to be estimated if data were not documented by the facility. Thirdly, our study provides an indication of how costs might vary across facilities in rural areas, but because of the small sample size we were unable to confirm these statistically.

Nevertheless, this study provides detailed cost estimates following standard methodologies that add to the evidence base around HIV testing and treatment. It also identified patterns of resource use related to the different services, indicating a re-thinking of resource allocation at the facility level maybe required to improve efficiency and reduce the unit cost of HIV testing. The sampled facilities are typical of rural health facilities in Malawi and the prices of important contributors to cost are set at the national level, suggesting our estimates are a good basis for making cost estimates elsewhere in the country. Finally, this study was completed before the Covid 19 pandemic which may have implications for achieving the UNAIDS targets.

## Conclusion

Moving towards differentiated testing strategies in this setting can lead to efficiency improvements and therefore, freeing up resources for achieving the 90–90-90 goals. Among the recommendations is the increased sharing of personnel across activities at facilities. This study also highlights the importance of preserving the life-span of first-line regimens and therefore, underlines the need for higher coverage of regular viral load monitoring. The results show that these types of cost analysis continue to be a useful tool in identifying areas for informing planning, achieving efficiency and freeing up resources in an era of stagnating funds.

## Supplementary information

**Additional file 1.** : Supplementary Tables.

## Data Availability

Data will be available for the next five years upon request to the study PI: Alison.Wringe@lshtm.ac.uk

## Abbreviations

ART: Antiretroviral therapy
FTE: Full time equivalent
HDSS: Health and demographic surveillance site
HMIS: Health management information system
M&E: Monitoring and evaluation
MoH: Ministry of Health
PLHIV: People living with HIV
US$: United States Dollars
WHO: World Health Organization
