# Supplementary material for: Assessing the costs and efficiency of HIV testing and treatment services in rural Malawi: implications for future “test and start” strategies
Source: BMC Health Serv Res. 2020 Aug 12;20:740. doi: 10.1186/s12913-020-05446-5 (PMC7422472; doi:10.1186/s12913-020-05446-5)
Supplement: Supplementary file 1 — Additional file 1. : Supplementary Tables. [file 12913_2020_5446_MOESM1_ESM.docx]

Table s1: Previous cost studies in Malawi

| **Authors** | **Study sites (sample; cost perspective; dates)** | **HIV service costed** | **Resource inputs costed** | **Outcome measured** | **Outcome*** |
| --- | --- | --- | --- | --- | --- |
| Mwenge | 15 Public rural primary health | Facility-based | Personnel | Number of HIV tests | 2359 |
| et al. | facilities from Blantyre; Machinga; | testing | HIV rapid test kits (RTK) | HIV prevalence | 9% |
| *2017* | Mwanza; and Neno Districts |  | General supplies e.g. Registers | Testing episodes per staff | 1132 (range: 519–2075) |
|  | Perspective: health provider |  | Overheads | Total annual costs | $14,822 (range: $5,386-$25,124) |
|  | *June 2016 - April 2017* |  | Buildings (space); equipment | Mean cost per test | $4.92 (range: $2.95-$8.33) |
|  |  |  | Vehicles | Mean cost per HIV case detected | $79.58 (range: 26.45-215.44) |
|  |  |  |  |  |  |
| Maheswaran | 3 health facilities (1 Central | Facility-based | Consumables e.g. ST kits & RTK | HIV prevalence | Facility 11.2%-31.5%; ST 9.0% |
| et al. | hospital; 2 PHC) from Blantyre | testing | Personnel | Facility per individual tested | US$7.53 (INT$20.25), US$10.57 |
| *2016* | District & 1241 individuals from | Self-testing (ST) | Training |  | (INT$25.18), US$8.90 (INT$20.44) |
|  | 3 urban suburbs |  | M&E: community; facility; district | HIVST per individual tested | US$8.78 (INT$17.25). |
|  | Perspective: Provider |  | Overheads (facility-based testing only) | Facility per HIV case identified | US$28.30-US$76.14 |
|  | *February 2013 - April 2014* |  | Buildings (facility-based testing only) | HIVST per HIV case identified | US$97.50 |
|  |  |  |  |  |  |
| Tagar et al. | 30 randomly selected | ART | Medication ART & Opportunistic Infections | Mean cost of delivering ART (per patient-year) | $136 (95% CI $119–$154) |
| *2014* | (national) health facilities |  | Personnel (direct & indirect) |  |  |
|  | Perspective: Payer (government |  | Clinical & non-clinical supplies |  |  |
|  | or donor)  *2010 - 2011* |  | Laboratory costs incl. outside facility |  |  |
|  |  |  | Adherence & other support programs |  |  |
|  |  |  | incurred at facility e.g community health workers |  |  |
|  |  |  | Nutritional support |  |  |
|  |  |  | Training |  |  |
|  |  |  | Equipment |  |  |
|  |  |  | Building maintenance; admin support |  |  |
|  |  |  |  |  |  |

* Costs adjusted to 2016 United States dollars (US$) (Mwenge et al. 2017); 2014 US$ (Maheswaren et al. 2016; US$ year not specified in Tagar et al. 2014

Table s2: Description of HIV service activities in Malawi [Source MoH, Malawi HIV Testing services guidelines 2016; MoH, Malawi Integrated Clinical HIV Guidelines. 3rd ed; 2016]

| **Service** | **Summary of Service** | **Core service activities** | **Description of core activities at facility level** | **Description of core activities at district and national level** |
| --- | --- | --- | --- | --- |
| **HIV testing** | Two approaches to facility-based HIV testing: 1) voluntary counselling and testing where clients voluntarily seek to know HIV status; 2) provider initiated testing and counselling provided in in-inpatient ward (adult & paediatric); ANC; NRU; Maternity; tuberculosis & sexually transmitted infections clinics, and all other out-patient. ALL clients must consent to be tested and informed on patient confidentiality/ disclosure and are reminded they can decline testing. ALL test results (incl. ANC, out-patient etc.) are recorded in a national register. | Pre-test counselling | All clients pre-tested are provided with information on HIV and testing. Messages include the importance of HIV diagnosis, the testing process, HIV risks and reduction, and the meaning of a positive and a negative diagnosis. For provider initiated clients, contextual pre-test information is provided e.g. risk of co-infection to tuberculosis patients; risk of HIV transmission to infants from pregnant women. | **Quality assurance for counselling:** Testing supervisors are expected to conduct quarterly site visits to: provide supportive supervision; ensure minimum standards; conduct client exit interviews; sit-in observations; provide remediation (corrective measures); and conduct testing provider meetings with documentation of minutes. **Quality assurance for testing:** District laboratory supervisors conduct site supervision every month, which include stock checks, and the national supervision team every quarter. Quality Assurance Reference Laboratory technicians provide on-site quality control including supervision and proficiency testing. **Surveillance / auditing Monitoring & Evaluation** |
|  |  | HIV testing | HIV Antibody rapid (finger-prick) test done in designated testing and counselling room or in the ART clinic if there is a testing room. 1st test conducted (Determine)-if non-reactive patient is reported as HIV negative, if reactive confirmatory test (Uni-gold) undertaken immediately by a different counsellor. If 2nd test reactive, patient is reported as HIV positive, if non-reactive test is recorded as discordant. If discordant a repeat test undertaken using 1st and 2nd tests - if tests remain discordant patient is retested after 4 weeks, and if 3rd time discordant dried blood spot (DBS) collected and sent to central laboratory for DNA-PCR diagnosis. |  |
|  |  | Post HIV Counselling | Post-test counselling provided to all tested regardless of outcome. Post-test messaging depends on whether test is negative when client is informed on risk reduction etc.; or positive when client is offered to be initiated on ART. |  |
| **HIV care and treatment** | Only MOH-certified clinical ART providers can prescribe ART (e.g. medical doctors; clinical officers; medical assistants; registered nurses; nurse midwife/technicians). Only health workers and qualified pharmacy personnel are allowed to dispense ARVs. | Confirmatory test | All patients require a confirmatory HIV test (1st and 2nd tests) before initiating on ART. If result is reactive (both 1st and 2nd tests) then patient is recorded as confirmed positive. If discordant then tests are repeated, and if repeat tests also discordant then DBS collected and sent to reference lab. Infants/children < 24 months require a confirmatory DNA-PCR a second DBS sample collected before or on the same day as starting ART. |  |
|  |  | Counselling | All patients asked to attend group counselling when "key facts" are shared e.g. viral load monitoring. All newly initiating patients are counselled individually on: importance of ART adherence; ART dosage; possible side effects and signs to look out for; and arrangements for next appointment. . |  |
|  |  | Starting ART | Patients >35kg are started on regimen 5A immediately after confirmatory tests or after 2 weeks (if patient reflection time needed). Patients <35kg are started on regimen 2P. All adults 30+ are screened for hypertension. |  |
|  |  | Monitoring | At every visit patients are checked for: HIV-related diseases; ART treatment failure; and ART drug side effects. These are checked from nutritional assessments (e.g. checks for wasting), and from clinical symptoms against a clinical monitoring list. e.g. tuberculosis symptoms include weight loss. Patients also assessed for ART adherence. If poor adherence suspected then intensive adherence counselling provided. |  |
|  |  | Preventive services for HIV patients | Includes a package designed to reduce HIV transmission from HIV patients to their sexual partners and mother to child transmission. Services include: family planning (e.g. provision of condoms); Cotrimoxazole; HIV exposed and infected infants from 6 weeks; HIV infected adults - Cotrimoxazole for life for all HIV infected persons; Isoniazid preventive therapy in high tuberculosis burden areas; and a treated net to each patient enrolled into HIV care every 2 years. Early infant - Nevirapine syrup up to 6 weeks, Cotrimoxazole to 24 months - testing 6 weeks DNA-PCR; 12 & 24 months rapid test. |  |
|  |  | Collection of DBS | For early infant diagnosis and for viral load - Routine viral load currently being scaled up (2016 guidelines) - currently suggested at 6 months; then every 2 years if patient is adherent. |  |

Table s3: Standard 1^st^ line and 2^nd^ line ART formulations in Malawi [Source MoH, Malawi Integrated Clinical HIV Guidelines. 3rd ed; 2016 ]

| **ART** | **Long name** | **Short name** | **Formulation** | |
| --- | --- | --- | --- | --- |
|  |  |  | **Adult** | **Child** |
| 1st line | Tenofovir/Lamivudine/Efavarinz | TDF/3TC/EFV | 300/300/600 |  |
|  | Zidovudine/Lamivudine/Nevarapine | AZT/3TC/NVP | 300/150/200 | 60/30/50 |
|  | Abacavir/Lamivudine+Nevarapine | ABC/3TC+NVP | 600/300+200 | 60/30+50 |
|  | Zidovudine/Lamivudine+Efavarinz | AZT/3TC+EFV | 300/150+600 | 60/30+200 |
|  | Tenofovir/Lamivudine+Nevarapine | TDF/3TC+NVP | 300/300+200 |  |
|  |  |  |  |  |
| 2nd line | Tenofavir/Lamivudine+Atazanavir/Ritonavir | TDF/3TC+ATV/r | 300/300+300/100 |  |
|  | Zidovudine/Lamivudine+Atazanavir | AZT/3TC+ATV/r | 300/150+300/100 |  |
|  | Abacavir/Lamivudine+Lopinavir/Ritonavir | ABC/3TC+LPV/r | 600/300+200/50 | 60/30+100/25 |
|  |  |  |  |  |

Table s4: HIV testing services and HIV care and treatment staffing levels

|  | **N*** | **FTE**** | **Testing** |  | **N*** | **FTE**** | **Care & Treatment** |
| --- | --- | --- | --- | --- | --- | --- | --- |
| **Clinic A** | 3 | 0.6 | Health Surveillance Assistant |  | 2 | 0.2 | Nurse Aide |
|  | 1 | 0.4 | Nurse technician |  | 1 | 0.2 | Senior medical assistant |
| ***Total*** | ***4*** | ***1.0*** |  |  | ***3*** | ***0.4*** |  |
|  |  |  |  |  |  |  |  |
| **Clinic B** | 8 | 4.0 | Counsellor |  | 8 | 4.0 | Adherence counsellors |
|  | 1 | 0.0 | Other |  | 1 | 0.5 | Lab Assistant |
|  |  |  |  |  | 1 | 0.0 | Unidentified |
| ***Total*** | ***9*** | ***4.0*** |  |  | ***10*** | ***4.5*** |  |
|  |  |  |  |  |  |  |  |
| **Clinic C** | 2 | 2.0 | Volunteer counsellor |  |  | 0.5 | Adherence counsellor |
|  | 1 | 0.5 | Counsellor |  |  | 0.2 | Drug dispenser |
|  |  |  |  |  |  | 0.3 | Sen. medical assistant |
| ***Total*** | ***3*** | ***2.5*** |  |  | ***2*** | ***1.0*** |  |
|  |  |  |  |  |  |  |  |
| **Hospital D** | 5 | 3.0 | Counsellor |  | 4 | 2.0 | Adherence counsellors |
|  | 6 | 0.2 | Nurse / midwife |  | 1 | 0.2 | Clinical Officer |
|  | 2 | 1.0 | Health Surveillance Assistant |  | 1 | 0.2 | Lab technician |
|  |  |  |  |  | 4 | 0 | Unidentified |
| ***Total*** | ***13*** | ***4.2*** |  |  | ***10*** | ***2.4*** |  |
|  |  |  |  |  |  |  |  |
| **Hospital E** | 4 | 4.0 | Health Diagnostic Assistant |  | 5 | 1.25 | Midwife |
|  | 4 | 0.6 | Nurse/midwife technician |  | 1 | 0.25 | Clinical Officer |
|  |  |  |  |  | 1 | 0.4 | Drug dispenser |
|  |  |  |  |  | 4 | 2.0 | Adherence counsellors |
|  |  |  |  |  | 1 | 0.5 | Lab technician |
|  |  |  |  |  | 5 | 0.0 | Unidentified |
| ***Total*** | ***8*** | ***4.6*** |  |  | ***17*** | ***4.4*** |  |
|  |  |  |  |  |  |  |  |

* Number of staff reported in facility survey

** FTE full time equivalent estimated from observations in facility, staff interviews, and staff timesheets

Table s5: Annual HIV positivity rate by facility characteristic

|  | **No. of tests October 2016 – September 2017** | | | |
| --- | --- | --- | --- | --- |
|  | **Determine (1^st^ test)** | **Unigold (2^nd^ test & confirmatory)** | **New positives** | **HIV positivity rate** |
| Clinic | 5,580 | 268 | 129 | 2.3% |
| Hospital | 12,929 | 705 | 324 | 2.5% |
| Public | 14,604 | 795 | 350 | 2.4% |
| Faith-based | 3,905 | 178 | 103 | 2.6% |
| Remote rural | 2,436 | 90 | 55 | 2.3% |
| Rural | 16,073 | 883 | 398 | 2.5% |
